# Molecular Characterization of *bla*_*IMP*__–__*4*_-Carrying *Enterobacterales* in Henan Province of China

**DOI:** 10.3389/fmicb.2021.626160

**Published:** 2021-02-17

**Authors:** Wentian Liu, Huiyue Dong, Tingting Yan, Xuchun Liu, Jing Cheng, Congcong Liu, Songxuan Zhang, Xiang Feng, Luxin Liu, Zhenya Wang, Shangshang Qin

**Affiliations:** ^1^School of Pharmaceutical Sciences, Zhengzhou University, Zhengzhou, China; ^2^Key Laboratory of Advanced Drug Preparation Technologies, Ministry of Education, Zhengzhou University, Zhengzhou, China; ^3^Department of Medical Laboratory, Yicheng District Central Hospital, Zhumadian, China; ^4^Key Laboratory of “Runliang” Antiviral Medicines Research and Development, Institute of Drug Discovery and Development, Zhengzhou University, Zhengzhou, China

**Keywords:** *bla*_*IMP*__–__4_, transposon-like structure, class 1 integron, carbapenem-resistant *Enterobacterales*, N plasmid

## Abstract

Carbapenem-resistant *Enterobacterales* (CRE) pose a serious threat to clinical management and public health. We investigated the molecular characteristics of 12 IMP-4 metallo-β-lactamase-producing strains, namely, 5 *Enterobacter cloacae*, 3 *Escherichia coli*, 2 *Klebsiella pneumoniae*, and 2 *Citrobacter freundii*. These strains were collected from a tertiary teaching hospital in Zhengzhou from 2013 to 2015. The minimum inhibitory concentration (MIC) results showed that each *bla*_*IMP*__–__4_-positive isolate was multidrug-resistant (MDR) but susceptible to colistin. All of the *E. coli* belonged to ST167, two *C. freundii* isolates belonged to ST396, and diverse ST types were identified in *E. cloacae* and *K. pneumoniae*. S1-PFGE, Southern blotting, and PCR-based replicon typing assays showed that the *bla*_*IMP*__–__4_-carrying plasmids ranged from ∼52 to ∼360 kb and belonged to FII, FIB, HI2/HI2A, and N types. N plasmids were the predominant type (8/12, 66.7%). Plasmid stability testing indicated that the *bla*_*IMP*__–__4_-carrying N-type plasmid is more stable than the other types of plasmids. Conjugative assays revealed that three of the *bla*_*IMP*__–__4_-carrying N plasmids were transferrable. Complete sequence analysis of a representative N type (pIMP-ECL14–57) revealed that it was nearly identical to pIMP-FJ1503 (KU051710) (99% nucleotide identity and query coverage), an N-type *bla*_*IMP*__–__4_-carrying epidemic plasmid in a *C. freundii* strain. PCR mapping indicated that a transposon-like structure [IS*6100-mobC-intron (K1.pn.I3)-bla*_*IMP*__–__4_*-IntI1-*IS*26*] was highly conserved in all of the N plasmids. IS*26* involved recombination events that resulted in variable structures of this transposon-like module in FII and FIB plasmids. The *bla*_*IMP*__–__4_ gene was captured by a *sul1*-type integron In1589 on HI2/HI2A plasmid pIMP-ECL-13–46.

## Introduction

The Zn(II)-containing metallo-β-lactamases (MBLs) comprise Imipenemase (IMP), New Delhi metallo-β-lactamase (NDM), and Verona Integron-encoded Metallo-β-lactamase (VIM) types that belong to class B β-lactamase according to the Ambler classification. MBLs can hydrolyze nearly all β-lactams, including carbapenems, which are important antibiotics in clinical practice and the “last line” drugs for treating infections caused by multiple drug-resistant (MDR) Gram-negative bacteria (Boyd et al., 2020). The rapid spread of MBLs among *Enterobacterales* has led to the increased prevalence of carbapenem-resistant *Enterobacterales* (CRE), and this presents a challenge for infection treatment worldwide (Nordmann and Poirel, 2019). Unlike NDMs, IMP-type β-lactamases are not often detected in CRE from China (Zhang et al., 2017; Wang et al., 2018). The most commonly encountered *bla*_*IMP*__–__4_ gene has been found captured by class 1 integrons and carried by plasmids belonging to multiple replicon types including HI2, L/M, A/C, and N for dissemination (Lai et al., 2017; Matsumura et al., 2017). An epidemic N plasmid in *Enterobacterales* isolates was recently recovered from Shanghai, Guangdong, and Fujian provinces of China and was responsible for the dissemination of *bla*_*IMP*__–__4_ gene (Wang et al., 2017). It is not known if this type of plasmid is prevalent in other regions of China and if it is involved in the spread of *bla*_*IMP*_ genes. We conducted a retrospective study to investigate the prevalence and molecular characterization of IMP-positive *Enterobacterales* isolates in Henan Province within the north central region of China.

## Materials and Methods

### Bacterial Isolates and Antimicrobial Susceptibility Testing

From January 2013 to December 2015, a retrospective survey for MBLs in CRE isolated from a tertiary teaching hospital of Zhengzhou University identified 12 *bla*_*IMP*__–__4_ positive isolates, which were recovered from different types of clinical specimens (Table 1). The study and consent procedure was approved by the Ethical Committee of Zhengzhou University. PCR and sequencing were used to identify MBL encoding genes, including *bla*_*IMP*_, *bla*_*NDM*_, and *bla*_*VIM*_, as described previously (Doyle et al., 2012). Antimicrobial susceptibility of the 12 *bla*_*IMP*__–__4_-positive isolates and their transconjugants was determined using microbroth and agar dilution methods according to the Clinical and Laboratory Standards Institute (CLSI) guidelines (CLSI, 2019). *Escherichia coli* ATCC25922 was used as the quality control.

**TABLE 1:** Characteristics of *bla*_IMP__–__4_-positive CRE isolates.

**Isolate^a^**	**Clinical features**				**MLST^b^**	***bla*_IMP__–__4_-carrying plasmids**	
	**Age/sex**	**Specimen**	**Diagnosis/ward^c^**	**Outcome**		**Plasmid name**	**Type and size (kb)**
ECL-13–46	25 years/female	Urine	Multiple injury and lung infection/neurosurgery	Discharge	ST231	pIMP-ECL-13–46	HI2/HI2A/360
ECL-14–57	72 years/female	Blood	Viral encephalitis and lung infection/EICU	Discharge	ST754	pIMP-ECL-14–57	N/52
ECL-15–65	54 years/male	Wound	Arterial ischemia and thrombosis of right lower/vascular surgery	Discharge	ST97	pIMP-ECL-15–65	N/52
ECL-15–101	60 years/male	Urine	Prostatic hyperplasia with urinary retention/urology	Discharge	ST133	pIMP-ECL-15–101	N/52
ECL-15–284	45 years/male	Abdominal drainage	Severe acute pancreatitis/ICU	Death	ST133	pIMP-ECL-15–284	N/52
KP-13–9	6 months/male	Sputum	Lung infections and asphyxia/PICU	Discharge	ST14	pIMP-KP-13–9	FII/110
KP-15–285	6 months/male	Sputum	Severe pneumonia/PICU	Death	ST17	pIMP-KP-15–285	N/52
EC-13–25	78 years/female	Urine	Bronchiectasis/respiratory and sleep department	Discharge	ST167	pIMP-EC-13–25	N/52
EC-13–26	72 years/male	Sputum	ACVD/NSICU	Discharge	ST167	pIMP-EC-13–26	N/52
EC-14–52	58 years/female	Urine	Renal calculi/urology	Discharge	ST167	pIMP-EC-14–52	N/52
CF-15–288	19 years/female	CSF^*d*^	Cerebral hemorrhage/NSICU	Discharge	ST396	pIMP-CF-15–288	FIB/130
CF-15–127	26 years/male	CSF	Headache and dizziness/NSICU	Death	ST396	pIMP-CF-15–127	FIB/130

*^a^ECL, E. cloacae strains; EC, E. coli strains; KP, K. pneumoniae strains; CF, C. freundii strains.*

*^b^MLST, multilocus sequence typing; -, not detected.*

*^*c*^ICU, intensive care unit; NSICU, neuroscience ICU; EICU, emergency ICU; PICU, pediatric ICU; ACVD, acute cardiovascular disease.*

*^*d*^CSF, cerebrospinal fluid.*

### Bacterial Genotyping

Multilocus sequence typing (MLST) for *Klebsiella pneumoniae*, *Enterobacter cloacae*, *Citrobacter freundii*, and *E. coli* isolates were performed using previously described methods (Qin et al., 2014; Liu et al., 2015). The PCR products were purified and sequenced, and the allelic profiles and sequence types (STs) were assigned using online databases (https://pubmlst.org/ for *K. pneumoniae*, *E. cloacae*, and *C. freundii*, http://mlst.warwick.ac.uk/mlst/dbs/Ecoli for *E. coli*).

### Conjugation Assay, S1-PFGE, and Southern Blotting

Conjugation experiments were conducted using methods described previously at 25, 30, and 37°C. Briefly, the *bla*_*IMP*__–__4_-positive isolates served as the donor, while *E. coli* EC600 (rifampin resistant) was used as the recipient strain. Transconjugants were selected on Mueller–Hinton (MH) agar supplemented with sodium rifampin (200 μg/ml) and meropenem (2 μg/ml). The presence of the *bla*_*IMP*__–__4_ gene and other resistance genes in transconjugants was confirmed by PCR, DNA sequencing, and antimicrobial susceptibility. S1-PFGE and Southern blotting were conducted, according to published methods, to estimate sizes of *bla*_*IMP*__–__4_ plasmids (Qin et al., 2014).

### Plasmid Sequencing and Genetic Environments of *bla*_*IMP*__–__4_ Analysis

**FIGURE 1:**
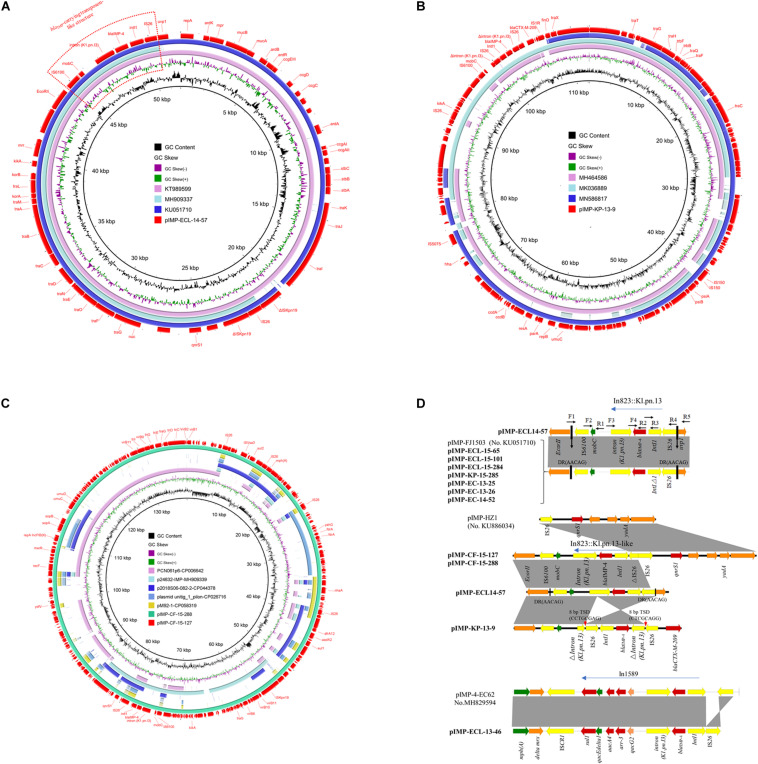
**(A)** Circular comparison between plasmid pIMP-ECL14-57 (MH727565, in this study) and other similar plasmids. Plasmid pIMP-ECL14-57 (the outer circle) was used by the BRIG software as a reference plasmid to perform the sequence alignment with BLASTN. The different colors indicated different plasmids and are listed in the color key. **(B)** Circular comparison between plasmid pIMP-KP-13-9 and other reported similar plasmids. Plasmid pIMP-KP-13-9 (the outer circle) was used by the BRIG software as a reference plasmid to perform the sequence alignment with BLASTN. The different colors indicate different plasmids and are listed in the color key. **(C)** Circular comparison between plasmids pIMP-CF-15-288 and pIMP-CF-15-127 and similar plasmids. Plasmids pIMP-CF-15-288 and pIMP-CF-15-127 (the outermost two circles) were used by the BRIG software as a reference plasmid to perform the sequence alignment with BLASTN. The different colors indicate different plasmids and are listed in the color key. **(D)** Comparison of the *bla*_*IMP*__–__4_ gene environments identified in this study with other publications: pIMP-FJ1503 (accession no. KU051710) and pIMP-4-EC62 (accession no. MH829594). Light gray shading indicated homologous regions (>99% DNA identity). Genetic contexts of class 1 integron carrying *bla*_*IMP*__–__4_ are shown. The different boxed arrows indicate the positions, directions of transcription, and predicted function of the genes. Positions of the primers used for PCR mapping are indicated by arrows. Genes, mobile elements, and other features are colored based on function classification.

The plasmids of the *bla*_*IMP*__–__4_-positive strains were extracted using the Qiagen Midi kit (Qiagen, Hilden, Germany) and transformed into *E. coli* DH5α by electroporation. Transformants were selected on Luria–Bertani (LB) agar plates containing meropenem (2 μg/ml), and we confirmed the presence of the *bla*_*IMP*__–__4_ gene by using PCR and sequencing. Plasmid replicons were determined using the PCR-based replicon typing method (Carattoli et al., 2005). Plasmids were sequenced based on the Illumina HiSeq2000 platform with 2 × 100 bp paired-end reads (Majorbio Company, Shanghai, China) and the Nanopore MinION (long-read) sequencing platform. The sequencing reads were assembled *de novo* using SOAP*denovo* v2.04. Open reading frame prediction and annotation were done with Glimmer 3.02^1^ and BLAST at NCBI^2^. Plasmid comparisons were performed using BRIG^3^ (Alikhan et al., 2011) and Easyfig^4^ tools (Sullivan et al., 2011). The complete sequence of the plasmids pIMP-ECL14-57, pIMP-KP-13-9, pIMP-CF-15-127, and pIMP-CF-15-288 and the ∼46 kb fragment from pIMP-ECL-13-46 were deposited in GenBank with accession nos. MH727565 (pIMP-ECL14-57), CP068028 (pIMP-KP-13-9), CP068026 (pIMP-CF-15-127), CP068027 (pIMP-CF-15-288), and (CP068240) (pIMP-ECL-13-46, partial sequence). The final dataset of pIMP-KP-13-9, pIMP-CF-15-127, and pIMP-CF-15-288 and the ∼46 kb fragment from pIMP-ECL-13-46 is available as a fasta file from Figshare; doi: 10.6084/m9.figshare.13515482^5^ The genetic environments surrounding the *bla*_*IMP*__–__4_ gene on the other seven N plasmids were investigated by PCR mapping and sequencing, and the plasmid pIMP-ECL14-57 and an HI2 plasmid pIMP4-SEM1 (KX810825) were used as references. PCR primers were designed from the reference sequences and are listed in Supplementary Table 1. The locations of the primers are shown in Figure 1D.

### Plasmid Stability

Stability tests for plasmids were conducted as described previously (Wang et al., 2017). Briefly, the *bla*_*IMP*__–__4_-harboring transformants from ECL14-57, CF-15-127, and ECL-13-46, which were representative of *bla*_*IMP*__–__4_-carrying N, F, and HI2 plasmids characterized in this study, respectively, were used as the test strains. The overnight growths of the bacteria in LB broth were inoculated into 2 ml of a fresh LB broth and incubated for 12 h at 37°C (time zero). The above process was repeated every 12 h (equivalent to 10 generations each). At time zero and after passage without antibiotic for 50, 100, 150, and 200 generations, a sample of the culture was diluted and spread onto a LB plate. One hundred colonies were picked and replica plated onto a pair of plain and antibiotic-containing (0.5 μg/ml meropenem) LB plates. Plasmid stability was determined by the percentage of colonies growing on the antibiotic-containing plates.

## Results

### Overview of the *bla*_*IMP*__–__4_-Positive Isolates

A total of 12 (12/317, 3.79%) *bla*_*IMP*__–__4_ positive isolates, namely, 5 *E. cloacae*, 3 *E. coli*, 2 *K. pneumoniae*, and 2 *C. freundii* strains, were obtained from 317 CRE. These strains were recovered from different sample types including urine, blood, wound, abdominal drainage, sputum, and cerebrospinal fluid (Table 1). Over half of the *bla*_*IMP*__–__4_-carrying isolates (7/12, 58.33%) were collected from the ICU department, and the mortality among the patients infected with a *bla*_*IMP*__–__4_-positive isolate was 25% (3/12) (Table 1). These patients were diagnosed with different clinical diseases and none of them had a history of foreign travel.

For the antimicrobial susceptibility profiles, all the *bla*_*IMP*__–__4_-positive isolates were susceptible to colistin [minimum inhibitory concentrations (MICs) of ≤2 μg/ml]; tigecycline also had high activity against these isolates (MIC_50_ = 0.5 μg/ml) (Table 2). Our observation is consistent with previous data from both China and other countries which showed that colistin and tigecycline are effective for the treatment of infections caused by CRE (Wang et al., 2018).

**TABLE 2:** Antibiotic susceptibilities of *bla*_*IMP*__–__4_-positive CRE and their transconjugants.

**Isolate^a^**	**Antibiotic susceptibility (μ g/ml) to^b^**															
	**IPM**	**MEM**	**ATM**	**CAZ**	**LVX**	**GEN**	**AMK**	**CHL**	**TET**	**TGC**	**CST**	**FOF**	**AMP**	**CFZ**	**CFX**	**TZP**
ECL-13-46	>64	>64	>64	>64	64	>64	>64	>64	>64	1	2	>1,024	ND	ND	ND	256
ECL-14-57	32	64	64	>64	64	64	16	>64	>64	4	1	64	ND	ND	ND	256
ECL-15-65	>64	64	64	>64	2	8	2	>64	8	1	0.5	<1	ND	ND	ND	>512
ECL-15-101	>64	>64	>64	>64	64	8	4	>64	8	4	1	<1	ND	ND	ND	>512
ECL-15-284	64	64	>64	>64	64	64	16	>64	8	0.25	1	64	ND	ND	ND	64
KP-13-9	64	64	64	>64	8	1	4	4	8	0.25	1	16	>256	>256	>256	128
KP-15-285	>64	>64	>64	>64	4	1	2	4	>64	0.5	0.5	16	>256	>256	>256	64
EC-13-25	8	16	>64	>64	64	64	>64	8	>64	0.25	0.5	2	>256	>256	>256	256
EC-13-26	4	4	64	64	16	>64	8	8	64	0.5	0.125	<1	>256	>256	>256	512
EC-14-52	4	16	64	64	0.5	>64	>64	4	64	0.25	0.5	16	>256	>256	>256	256
CF-15-288	64	64	>64	>64	4	8	8	8	4	2	1	4	ND	ND	ND	512
CF-15-127	64	32	64	>64	64	64	8	8	8	1	2	4	ND	ND	ND	256
Recipients																
*E. coli* EC600	0.25	<0.03	0.5	0.25	0.125	1	2	4	2	<0.125	0.5	<1	4	2	8	<2
Transconjugants																
ECL-15-101-EC600	16	8	0.25	>64	4	<0.25	4	4	1	0.5	0.5	< (<1	4	2	64	1
ECL-15-284-EC600	16	16	0.25	>64	4	<0.25	2	4	1	0.25	0.5	< (<1	8	2	64	2
KP-15-285-EC600	16	8	0.25	>64	1	<0.25	2	2	2	0.5	0.5	< (<1	4	2	32	1

*^a^All of the bla_*IMP*__–__4_-positive isolates were multidrug-resistant (MDR) strains. EC, E. coli strains; KP, K. pneumoniae strains; ECL, E. cloacae strains; CF, C. freundii strains. For the transconjugants, all were E. coli EC600 harboring plasmids from the respective clinical isolates.*

*^*b*^IPM, imipenem; MEM, meropenem; ATM, aztreonam; CAZ, ceftazidime; LVX, levofloxacin; GEN, gentamicin; AMK, amikacin; CHL, chloramphenicol; TET, tetracycline; TGC, tigecycline; CST, colistin; FOF, fosfomycin; AMP, ampicillin; CFZ, cefazolin; CFX, cefoxitin; TZP, piperacillin-tazobactam. ND, not determined (E. cloacae and C. freundii isolates are intrinsically resistant to AMP, CFZ, and CFX).*

### Bacterial Genotyping, Conjugation, and Plasmid Analysis

MLST was performed for all the IMP-4-positive *E. cloacae*, *E. coli*, *C. freundii*, and *K. pneumoniae* isolates. Based on the MLST results, five *E. cloacae* isolates were distributed to four ST types, namely, ST133 (*n* = 2), ST231 (*n* = 1), ST754 (*n* = 1), and ST97 (*n* = 1). All of the three *E. coli* isolates belonged to ST167, which is regarded as the most common clone of *E. coli* in China (Zhang et al., 2017; Wang et al., 2018). Two *C. freundii* isolates belonging to ST396. ST14- and ST17-type *K. pneumoniae* carried *bla*_*IMP*__–__4_ in this study (Table 1). Overall, the observation of diversity in the isolates of *E. cloacae* for carrying *bla*_*IMP*__–__4_ indicated that the mobile genetic elements, such as conjugative plasmids and transposons, might be responsible for the horizontal transfer of *bla*_*IMP*__–__4_ among different clones.

The *bla*_*IMP*__–__4_ gene was always carried by a plasmid, so S1-PFGE and Southern blotting were performed to identify *bla*_*IMP*__–__4_ harboring plasmids. The *bla*_*IMP*__–__4_ genes in all 12 CRE isolates were located on plasmids with sizes ranging from ∼52 to ∼360 kb. The ∼52 kb plasmids were predominant among those carrying *bla*_*IMP*__–__4_ (8/12, 66.7%). Conjugative assays revealed that only three ∼52 kb *bla*_*IMP*__–__4_-carrying plasmids were successfully transferred to *E. coli* EC600 from the donors by conjugation at frequencies of 3.2 × 10^–4^–4 × 10^–5^ per donor cell. The other nine IMP-4-encoding plasmids which failed to transfer to the recipient strain by conjugation were electrotransformed into *E. coli* DH5α. PCR-based replicon typing analysis for both transconjugants and transformants showed that all the ∼52 kb *bla*_*IMP*__–__4_-carrying plasmids were distributed in four *E. cloacae*, three *E. coli*, and one *K. pneumoniae* isolates belonging to plasmid replicon type N (Table 1 and Figure 1A). The details concerning plasmid name, size, and replicon type are summarized in Table 1.

### Sequence Analysis of *bla*_*IMP*__–__4_-Carrying Plasmids and Genetic Environments of *bla*_*IMP*__–__4_

A representative N-type *bla*_*IMP*__–__4_-carrying plasmid named pIMP-ECL14-57, which came from *E. cloacae* strain ECL14-57, had 51,795 bp, with an average GC content of 50.52%, encoding 54 predicted open reading frames (ORFs). It shared extensive similarity with pIMP-FJ1503 (99% nucleotide identity and query coverage) (KU051710), an N-type *bla*_*IMP*__–__4_-carrying plasmid in a carbapenem-resistant *C. freundii* strain CRE1503 isolated from Hong Kong (Figure 1A). Comparative genomic analysis between these two plasmids revealed only two differences: (1) the intact IS*kpn19* element downstream of *qnrS1* that was carried by pIMP-FJ1503 was inserted by an IS*26* element in pIMP-ECL14-57 and (2) the *Int1* gene immediately upstream of *bla*_*IMP*__–__4_ was complete in pIMP-ECL14-57 but was truncated in pIMP-FJ1503 (Figure 1A). Only two resistance genes, namely *bla*_*IMP*__–__4_ and *qnrS1*, conferring resistance to carbapenems and quinolones, respectively, were identified in each plasmid. The *bla*_*IMP*__–__4_ gene-associated class 1 integron In823 was carried by a transposon-like structure [IS*6100-mobC-intron (K1.pn.I3)-bla*_*IMP*__–__4_*-IntI1-*IS*26*] bracketed by two 5 bp direct repeats (DR: AACAG) inserted between the *EcorII* and *uvp1* genes. In addition, this *bla*_*IMP*__–__4_-carrying transposon-like structure was also identified in the other seven N plasmids by using PCR mapping and sequencing (Figure 1D).

The FII plasmid pIMP-KP-13-9 was 112,209 bp long with an average GC content of 51.19% and encoding 138 predicted ORFs (Figure 1B). This plasmid showed 98.94% nucleotide identity and 84% query coverage with pIMP1572 (MH464586), a plasmid carrying both *bla*_*IMP*__–__26_ and *tet(A)* variants (Yao et al., 2020). Different from the plasmid pIMP1572, a Tn*1721*-like transposon structure carrying the *tet*(A) variant which is responsible for tigecycline was absent in pIMP-KP-13-9. Interestingly, a 3,447 bp region comprising an IS*26*, *int1*, the *bla*_*IMP*__–__4_ gene, and Δ*intron (K1.pn.I3)* in the *bla*_*IMP*__–__4_-carrying transposon-like structure in N plasmids was reversed in pIMP-KP-13-9 due to IS*26-*mediated recombination indicated by the presence of target site duplications (TSD) of 8 bp (CCTGCGAG).

The two FIB plasmids pIMP-CF-15-127 and pIMP-CF-15-288 obtained from different ST396 *C. freundii* strains were nearly identical (96.85% nucleotide identity and 100% query coverage) (Figure 1C), both of which harbored 268 predicted ORFs. The backbone region of pIMP-CF-15-127/pIMP-CF-15-288 (∼57.6 kb) containing *repA* (replication), *umuCD* (SOS mutagenesis), *sopAB* (plasmid-partition), and partial type IV secretion system (T4SS) encoding gene cluster shared 51% query coverage and 99.06% nucleotide identity with PCN061p6 (CP006642) from an O9 *E. coli* strain. A partial, but not an intact, T4SS encoding region in the two plasmids could explain the lack of conjugation of FIB plasmids. A similar *bla*_*IMP*__–__4_-carrying transposon-like structure in N plasmids was also found in pIMP-CF-15-127/pIMP-CF-15-288, while the IS26 element located immediately downstream of the int1 gene was disrupted by a 6,689 bp pIMP-HZ1 (KU886034)-derived segment encompassing IS*26*, *qnrS1*, and multiple functional genes (Figure 1D).

Overall, the *bla*_*IMP*__–__4_-associated In823 flanked by IS*6100* and IS*26* in N plasmids was conserved, and IS*26* involved recombination events that resulted in variable structures of this transposon-like module in the FII and FIB plasmids. Analysis of a ∼46 kb *bla*_*IMP*__–__4_-carrying segment from the HI2/HI2A plasmid pIMP-ECL-13-46 (failure to obtain complete sequence by WGS) revealed that the *bla*_*IMP*__–__4_ gene was present in the *sul1*-type integron In1589, which was first identified in an HI2 plasmid pIMP-4-EC62 obtained from *E. cloacae* EC62 of swine origin (Zhu et al., 2019).

### Stability of *bla*_*IMP*__–__4_-Carrying Plasmids

Plasmid stability analysis revealed that the N-type plasmid pIMP-ECL14-57 in transformants from ECL14-57 could be maintained at 100% over 200 generations of multiplication in the absence of antibiotics. However, drastic loss of the F-type plasmid pIMP-CF-15-127 and HI2-type plasmid pIMP-ECL-13-46 in transformants from CF-15-127 and ECL-13-46 was observed after 50 generations of multiplication, with 35 and 3%, respectively, retaining the *bla*_*IMP*__–__4_-harboring plasmid after 150 generations. These results revealed that, among the plasmids carrying *bla*_*IMP*__–__4_, the N type is more stable than the F type and HI2 type.

## Discussion

The IMP-4-type MBL, first identified in clinical *Acinetobacter* spp. from Hong Kong (Chu et al., 2001), has spread to Australia but has not been frequently detected as KPC-2 and NDM among CRE in mainland China (Xiong et al., 2016). The incidence (3.79%) of IMP-4-producing *Enterobacterales* observed in the CRE of this study was comparable to that found in a recent nationwide survey of CRE (3.6%) (Wang et al., 2018). The *bla*_*IMP*__–__4_ gene was found in four species, namely, *E. cloacae*, *E. coli*, *K. pneumoniae*, and *C. freundii*, which are the most common species carrying *bla*_*IMP*_ genes (Wang et al., 2018). A report from Australia indicated that IMP-4 was the predominant MBL type among CRE, particularly in carbapenem-resistant *E. cloacae* (CRECL) (Sidjabat et al., 2015). Our previous study together with recent findings from China revealed the dominance of NDM-type MBL among CRECL; whether IMP-4 is the second most common MBL in CRECL needs further study (Liu et al., 2015; Jin et al., 2018).

All of the *bla*_*IMP*__–__4_ genes in this study were carried by plasmids with diverse replicons. These included HI2, N, F, and especially the predominant N plasmids. The N type is a broad host range plasmid that carries a variety of resistance determinants and shows resistance to extended-spectrum-β-lactams, sulfonamides, quinolones, aminoglycosides, tetracyclines, and streptomycin (Eikmeyer et al., 2012). N plasmids are also associated with the spread of carbapenem-resistant determinants, such as *bla*_*NDM*_ and *bla*_*KPC*_ (Poirel et al., 2011; Partridge et al., 2012; Eilertson et al., 2017; Jiang et al., 2017; Partridge et al., 2018; Schweizer et al., 2019). This type of plasmid was recently identified as an epidemic plasmid for carrying *bla*_*IMP*__–__4_ among *Enterobacterial* species in China (Lai et al., 2017; Wang et al., 2017), and it was responsible for horizontal transmission of *bla*_*IMP*__–__6_ among *Enterobacterales* from Japan (Yamagishi et al., 2020). Our findings are consistent with these studies and indicate the prevalence of N *bla*_*IMP*__–__4_-carrying epidemic plasmids among CRE in other regions of China. Additionally, FII plasmids, which are carriers of the *bla*_*KPC*_ gene in *K. pneumoniae* (Partridge et al., 2018; Yang et al., 2020), were found to carry the *bla*_*IMP*_ gene in this study. Association with these widespread types of plasmids may accelerate dissemination of *bla*_*IMP*_ genes among *K. pneumonia*.

Class 1 integrons are common vehicles for carrying the *bla*_*IMP*_ genes. Multiple *bla*_*IMP*_-harboring class 1 integrons with considerable cassette array diversity, such as In992, 1312 (*bla*_*IMP*__–__1_), In809, 823, 1456, 1460, 1589 (*bla*_*IMP*__–__4_), In722, 1321 (*bla*_*IMP*__–__6_), In73 (*bla*_*IMP*__–__8_), In687 (*bla*_*IMP*__–__14_), In1310, 1386 (*bla*_*IMP*__–__26_), and 1385 (*bla*_*IMP*__–__38_), were identified in *Enterobacterales* and non-fermenting Gram-negative bacilli including *Pseudomonas aeruginosa* and *Acinetobacter* spp. (Lee et al., 2017; Matsumura et al., 2017; Papagiannitsis et al., 2017; Wang et al., 2017; Dolejska et al., 2018; Zhan et al., 2018; Zhu et al., 2019). Among these, the *bla*_*IMP*__–__4_-carrying In823 integron was the most frequently detected structure on N-type plasmids in isolates recovered from different regions of China including Henan Province (Feng et al., 2016; Wang et al., 2017).

## Conclusion

In conclusion, we determined the prevalence and molecular characterization of *bla*_*IMP*__–__4_-positive *Enterobacterales* in clinical specimens collected at a teaching hospital in Henan Province. Previously reported epidemic N-type plasmids exhibited superior stability compared with F- and HI2-type plasmids. N-type plasmids were the predominant plasmids carrying *bla*_*IMP*__–__4_ among the collected *Enterobacterales*. Associated with self-transmissible N plasmids, widespread FII plasmids and a successful epidemic *E. coli* ST167 clone might facilitate further dissemination of *bla*_*IMP*__–__4_ among the *Enterobacterales*. Surveillance is needed to monitor the spread of *bla*_*IMP*__–__4_-harboring *Enterobacterales*.

## Data Availability Statement

The datasets presented in this study can be found in online repositories. The names of the repository/repositories and accession number(s) can be found below: https://www.ncbi.nlm.nih.gov/, MH727565, CP068028, CP068026, CP068027, and CP068240.

## Ethics Statement

The studies involving human participants were reviewed and approved by the Ethical Committee of Zhengzhou University. Written informed consent to participate in this study was provided by the participants’ legal guardian/next of kin. Written informed consent was obtained from the individual(s), and minor(s)’ legal guardian/next of kin, for the publication of any potentially identifiable images or data included in this article.

## Author Contributions

SQ and ZW designed the study. WL, TY, CL, and SZ performed the experiments. HD, JC, LL, and XF analyzed the bioinformatics data. SQ and JC wrote the manuscript. All authors contributed to manuscript revision, read, and approved the submitted version.

## Conflict of Interest

The authors declare that the research was conducted in the absence of any commercial or financial relationships that could be construed as a potential conflict of interest.

## Footnotes

1 http://cbcb.umd.edu/software/glimmer/

2 https://blast.ncbi.nlm.nih.gov/Blast.cgi

3 http://brig.sourceforge.net/

4 http://mjsull.github.io/Easyfig/

5 https://figshare.com/s/a1dc76cc2fa6dd00d1b4

## References

[B1] Alikhan N. F. Petty N. K. Ben Zakour N. L. Beatson S. A. (2011). BLAST Ring Image Generator (BRIG): simple prokaryote genome comparisons. *BMC Genomics* 12:402. 10.1186/1471-2164-12-402 21824423 PMC3163573

[B2] Boyd S. E. Livermore D. M. Hooper D. C. Hope W. W. (2020). Metallo-beta-lactamases: structure, function, epidemiology, treatment options, and the development pipeline. *Antimicrob. Agents Chemother.* 64:e00397-20. 10.1128/aac.00397-20 32690645 PMC7508574

[B3] Carattoli A. Bertini A. Villa L. Falbo V. Hopkins K. L. Threlfall E. J. (2005). Identification of plasmids by PCR-based replicon typing. *J. Microbiol. Methods* 63 219–228. 10.1016/j.mimet.2005.03.018 15935499

[B4] Chu Y. W. Afzal-Shah M. Houang E. T. S. Palepou M. F. I. Lyon D. J. Woodford N. (2001). IMP-4, a novel Metallo-β-lactamase from nosocomial *Acinetobacter* spp. Collected in Hong Kong between 1994 and 1998. *Antimicrob. Agents Chemother.* 45 710–714. 10.1128/aac.45.3.710-714.2001 11181348 PMC90361

[B5] CLSI (2019). *Performance Standards for Antimicrobial Susceptibility Testing; 29th Informational Supplement.* CLSI Document M100. Wayne, PA: CLSI.

[B6] Dolejska M. Papagiannitsis C. Medvecky M. Davidova-Gerzova L. Valcek A. J. A. (2018). Characterization of the complete nucleotide sequences of IMP-4-encoding plasmids, belonging to diverse inc families, recovered from *Enterobacteriaceae* isolates of wildlife origin. *Chemotherapy* 62:e02434-17. 10.1128/aac.02434-17 29483121 PMC5923110

[B7] Doyle D. Peirano G. Lascols C. Lloyd T. Church D. L. Pitout J. D. D. (2012). Laboratory detection of *Enterobacteriaceae* that produce carbapenemases. *J. Clin. Microbiol.* 50 3877–3880. 10.1128/jcm.02117-12 22993175 PMC3503014

[B8] Eikmeyer F. Hadiati A. Szczepanowski R. Wibberg D. Schneiker-Bekel S. Rogers L. M. (2012). The complete genome sequences of four new IncN plasmids from wastewater treatment plant effluent provide new insights into IncN plasmid diversity and evolution. *Plasmid* 68 13–24. 10.1016/j.plasmid.2012.01.011 22326849

[B9] Eilertson B. Chen L. Chavda K. D. Kreiswirth B. N. (2017). Genomic characterization of two KPC-producing klebsiella isolates collected in 1997 in New York City. *Antimicrob. Agents Chemother.* 61:e02458-16. 10.1128/AAC.02458-16 28167551 PMC5365676

[B10] Feng W. Zhou D. Wang Q. Luo W. Zhang D. Sun Q. (2016). Dissemination of IMP-4-encoding pIMP-HZ1-related plasmids among *Klebsiella pneumoniae* and *Pseudomonas aeruginosa* in a Chinese teaching hospital. *Sci. Rep.* 6:33419. 10.1038/srep33419 27641711 PMC5027574

[B11] Jiang X. Yin Z. Yin X. Fang H. Sun Q. Tong Y. (2017). Sequencing of blaIMP-carrying IncN2 plasmids, and comparative genomics of IncN2 plasmids harboring class 1 integrons. *Front. Cell Infect. Microbiol.* 7:102. 10.3389/fcimb.2017.00102 28424761 PMC5371602

[B12] Jin C. Zhang J. Wang Q. Chen H. Wang X. Zhang Y. (2018). Molecular characterization of carbapenem-resistant *Enterobacter cloacae* in 11 chinese cities. *Front. Microbiol.* 9:1597. 10.3389/fmicb.2018.01597 30065717 PMC6056727

[B13] Lai K. Ma Y. Guo L. An J. Ye L. Yang J. (2017). Molecular characterization of clinical IMP-producing *Klebsiella pneumoniae* isolates from a Chinese Tertiary Hospital. *Ann. Clin. Microbiol. Antimicrob.* 16:42. 10.1186/s12941-017-0218-9 28629366 PMC5474851

[B14] Lee J. H. Bae I. K. Lee C. H. Jeong S. (2017). Molecular characteristics of first IMP-4-producing *Enterobacter cloacae* sequence type 74 and 194 in Korea. *Front. Microbiol.* 8:2343. 10.3389/fmicb.2017.02343 29326660 PMC5741837

[B15] Liu C. Qin S. Xu H. Xu L. Zhao D. Liu X. (2015). New Delhi metallo-beta-lactamase 1(NDM-1), the dominant carbapenemase detected in carbapenem-resistant *Enterobacter cloacae* from henan province, China. *PLoS One* 10:e0135044. 10.1371/journal.pone.0135044 26263489 PMC4532496

[B16] Matsumura Y. Peirano G. Motyl M. R. Adams M. D. Chen L. Kreiswirth B. (2017). Global molecular epidemiology of IMP-producing *Enterobacteriaceae*. *Antimicrob. Agents Chemother.* 61:e02729-16. 10.1128/aac.02729-16 28167555 PMC5365671

[B17] Nordmann P. Poirel L. (2019). Epidemiology and diagnostics of carbapenem resistance in gram-negative bacteria. *Clin. Infect. Dis.* 69 521–528. 10.1093/cid/ciz824 31724045 PMC6853758

[B18] Papagiannitsis C. Kutilova I. Medvecky M. Hrabak J. Dolejska M. J. A. (2017). Characterization of the complete nucleotide sequences of IncA/C plasmids carrying In809-like integrons from *Enterobacteriaceae* isolates of wildlife origin. *Chemotherapy* 61:e01093-17. 10.1128/aac.01093-17 28696228 PMC5571335

[B19] Partridge S. R. Kwong S. M. Firth N. Jensen S. O. (2018). Mobile genetic elements associated with antimicrobial resistance. *Clin. Microbiol. Rev.* 31:e00088-17. 10.1128/CMR.00088-17 30068738 PMC6148190

[B20] Partridge S. R. Paulsen I. T. Iredell J. R. (2012). pJIE137 carrying blaCTX-M-62 is closely related to p271A carrying blaNDM-1. *Antimicrob. Agents Chemother.* 56 2166–2168. 10.1128/AAC.05796-11 22252811 PMC3318322

[B21] Poirel L. Bonnin R. A. Nordmann P. (2011). Analysis of the resistome of a multidrug-resistant NDM-1-producing *Escherichia coli* strain by high-throughput genome sequencing. *Antimicrob. Agents Chemother.* 55 4224–4229. 10.1128/AAC.00165-11 21746951 PMC3165296

[B22] Qin S. Fu Y. Zhang Q. Qi H. Wen J. G. Xu H. (2014). High incidence and endemic spread of NDM-1-positive *Enterobacteriaceae* in Henan Province, China. *Antimicrob. Agents Chemother.* 58 4275–4282. 10.1128/AAC.02813-13 24777095 PMC4136005

[B23] Schweizer C. Bischoff P. Bender J. Kola A. Gastmeier P. Hummel M. (2019). Plasmid-mediated transmission of KPC-2 carbapenemase in *Enterobacteriaceae* in critically Ill patients. *Front. Microbiol.* 10:276. 10.3389/fmicb.2019.00276 30837980 PMC6390000

[B24] Sidjabat H. E. Townell N. Nimmo G. R. George N. M. Robson J. Vohra R. (2015). Dominance of IMP-4-Producing *Enterobacter cloacae* among carbapenemase-producing *Enterobacteriaceae* in Australia. *Antimicrob. Agents Chemother.* 59 4059–4066. 10.1128/aac.04378-14 25918153 PMC4468659

[B25] Sullivan M. J. Petty N. K. Beatson S. A. (2011). Easyfig: a genome comparison visualizer. *Bioinformatics* 27 1009–1010. 10.1093/bioinformatics/btr039 21278367 PMC3065679

[B26] Wang Q. Wang X. Wang J. Ouyang P. Jin C. Wang R. (2018). Phenotypic and genotypic characterization of carbapenem-resistant *Enterobacteriaceae*: data From a longitudinal large-scale CRE Study in China (2012-2016). *Clin. Infect. Dis.* 67 S196–S205. 10.1093/cid/ciy660 30423057

[B27] Wang Y. Lo W.-U. Lai R. W.-M. Tse C. W.-S. Lee R. A. Luk W.-K. (2017). IncN ST7 epidemic plasmid carrying blaIMP-4 in *Enterobacteriaceae* isolates with epidemiological links to multiple geographical areas in China. *J. Antimicrob. Chemother.* 72 99–103. 10.1093/jac/dkw353 27609049

[B28] Xiong J. Deraspe M. Iqbal N. Ma J. Jamieson F. B. Wasserscheid J. (2016). Genome and plasmid analysis of blaIMP-4-carrying *Citrobacter freundii* B38. *Antimicrob. Agents Chemother.* 60 6719–6725. 10.1128/AAC.00588-16 27572407 PMC5075078

[B29] Yamagishi T. Matsui M. Sekizuka T. Ito H. Fukusumi M. Uehira T. (2020). A prolonged multispecies outbreak of IMP-6 carbapenemase-producing *Enterobacter*ales due to horizontal transmission of the IncN plasmid. *Sci. Rep.* 10:4139. 10.1038/s41598-020-60659-2 32139745 PMC7057946

[B30] Yang X. Dong N. Chan E. W.-C. Zhang R. Chen S. (2020). Carbapenem resistance-encoding and virulence-encoding conjugative plasmids in *Klebsiella pneumoniae. Trends Microbiol.* 29 65–83. 10.1016/j.tim.2020.04.012 32448764

[B31] Yao H. Cheng J. Li A. Yu R. Zhao W. Qin S. (2020). Molecular characterization of an IncFIIk plasmid co-harboring blaIMP-26 and tet(A) variant in a clinical *Klebsiella pneumoniae* isolate. *Front. Microbiol.* 11:1610. 10.3389/fmicb.2020.01610 32793144 PMC7393768

[B32] Zhan Z. Hu L. Jiang X. Zeng L. Feng J. Wu W. (2018). Plasmid and chromosomal integration of four novel blaIMP-carrying transposons from *Pseudomonas aeruginosa*, *Klebsiella pneumoniae* and an *Enterobacter* sp. *J. Antimicrob. Chemother.* 73 3005–3015. 10.1093/jac/dky288 30351436

[B33] Zhang R. Liu L. Zhou H. Chan E. W. Li J. Fang Y. (2017). Nationwide surveillance of clinical carbapenem-resistant *Enterobacteriaceae* (CRE) strains in China. *Ebiomedicine* 19 98–106. 10.1016/j.ebiom.2017.04.032 28479289 PMC5440625

[B34] Zhu Y. Zhang W. Schwarz S. Wang C. Liu W. Chen F. (2019). Characterization of a blaIMP-4-carrying plasmid from *Enterobacter cloacae* of swine origin. *J. Antimicrob. Chemother.* 74 1799–1806. 10.1093/jac/dkz107 30879063

