# Supplementary figures and images for: Molecular Characterization of blaIMP–4-Carrying Enterobacterales in Henan Province of China
Source: Front Microbiol. 2021 Feb 17;12:626160. doi: 10.3389/fmicb.2021.626160 (PMC7925629; doi:10.3389/fmicb.2021.626160)

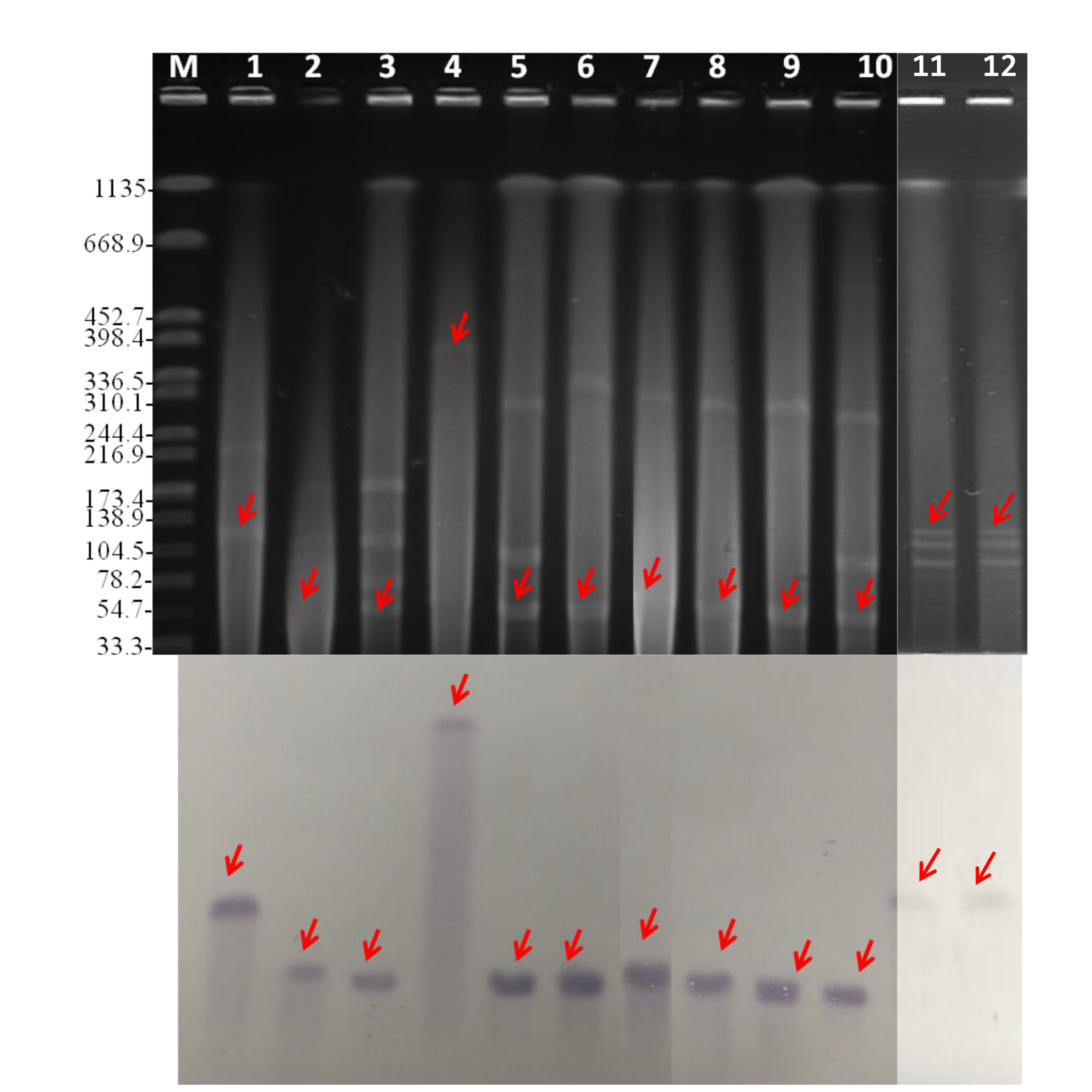

Supplement: Supplementary file 1 [file Image_1.TIF]
