# Supplementary material for: Molecular Characterization of blaIMP–4-Carrying Enterobacterales in Henan Province of China
Source: Front Microbiol. 2021 Feb 17;12:626160. doi: 10.3389/fmicb.2021.626160 (PMC7925629; doi:10.3389/fmicb.2021.626160)
Supplement: Supplementary file 2 [file Table_1.DOCX]

**SUPPLEMENTARY MATERIAL**

**Supplementary Table 1.** PCR primers and conditions used in this study.

| Primers | Sequence (5'-3') | Annealing  Temperature (℃) | Product  Size(bp) |
| --- | --- | --- | --- |
| F1 | TCAGTTCACGGGTATGGTT | 55 | 1692 |
| R1 | TTTGTGGCTCATTAGGTCA |  |  |
| F2 | CACCGGCTCCAGGTCTTA | 54 | 3194 |
| R2 | GCAGCAACGATGTTACGC |  |  |
| F3 | TCGCCGAGTGCGTATGTG | 55 | 2088 |
| R3 | CGCCTGGTAAGCAGAGTTTT |  |  |
| F4 | TTTATAGCCACGTTCCACA | 54 | 1880 |
| R4 | GCAAATACGGCATCAGTTA |  |  |
| F5 | TAGCCGTAAATGGAGTGTC | 54 | 2248 |
| R5 | TTCAGGTCGTCGAGTAAAG |  |  |
| F6 | GAAGAACCGCACAATCTCG | 55 | 2461 |
| R6 | GGCTGCCTGAAAGGAAAA |  |  |
| F7 | TTAGCTTGAACCTTACCGTCTT | 55 | 954 |
| R7 | AAACGATGCTCGCCTTCC |  |  |

**supplemental Figure S1**. Detection of *bla*_IMP-4_-carrying plasmids by S1-PFGE and Southern hybridization. S1-PFGE (top) and Southern blotting (bottom) with *bla*_IMP-4_ specific probe. Lane M, marker (*Salmonella* H9812); lane 1, KP-13-9; lane 2, EC-13-25; lane 3, EC-13-26; lane 4, ECL-13-46; lane 5, EC-14-52; lane 6, ECL-14-57; lane 7, ECL-15-65; lane 8, ECL-15-101; lane 9, ECL-15-284; lane 10, KP-15-285; lane 11, CF-15-288; lane 12, CF-15-127. The arrows indicate the plasmids positive for *bla*_IMP-4_.
